# Associations between sociodemographic factors and receiving "ask and advise" services from healthcare providers in India: analysis of the national GATS-2 dataset

**DOI:** 10.1186/s12889-022-14538-2

**Published:** 2022-11-18

**Authors:** Shoba Ramanadhan, Ziming Xuan, Jasmin Choi, Sitara L. Mahtani, Sara Minsky, Himanshu Gupte, Gauri Mandal, Dinesh Jagiasi, Kasisomayajula Viswanath

**Affiliations:** 1 Harvard T.H. Chan School of Public Health, 677 Huntington Avenue, 7th Floor, Boston, MA 02115 USA; 2 Boston University School of Public Health, 801 Massachusetts Ave Crosstown Center, Boston, MA 02118 USA; 3 Dana-Farber Cancer Institute, 450 Brookline Ave, LW 6th floor, Boston, MA 02215 USA; 4 Narotam Sekhsaria Foundation, 1st Floor, Nirmal Building, Nariman Point, Churchgate, Mumbai, 400 021 India; 5 Salaam Bombay Foundation, 1st Floor, Nirmal Building, Nariman Point, Churchgate, Mumbai, 400 021 India

**Keywords:** Tobacco, India, Brief advice, Equity, Inequities

## Abstract

**Background:**

India is home to about 12% of the world's tobacco users, with about 1.35 million tobacco-related deaths each year. The morbidity and mortality rates are socially patterned based on gender, rural vs. urban residence, education, and other factors. Following the World Health Organization's guidance, it is critical to offer tobacco users support for cessation as a complement to policy and environmental changes. Such guidance is typically unavailable in low-resource systems, despite the potential for population-level impact. Additionally, service delivery for tobacco control tends to be patterned by sociodemographic factors. To understand current activity in this area, we assessed the percentage of daily tobacco users being asked about tobacco use and advised to quit by a healthcare provider. We also examined social patterning of receipt of services (related to by rural vs. urban residence, age, gender, education, caste, and wealth).

**Methods:**

We analyzed cross-sectional data from India's 2016-2017 Global Adult Tobacco Survey (GATS-2), a nationally representative survey. Among 74,037 respondents, about 25% were daily users of smoked and/or smokeless tobacco. We examined rates of being asked and advised about tobacco use overall and based on rural vs. urban residence, age, gender, education, caste, and wealth. We also conducted multivariate logistic regression to assess the association of demographic and socioeconomic conditions with participants' receipt of “ask and advise” services.

**Results:**

Nationally, among daily tobacco users, we found low rates of individuals reporting being asked about tobacco use or advised to quit by a healthcare provider (22% and 19%, respectively). Being asked and advised about tobacco use was patterned by age, gender, education, caste, and wealth in our final regression model.

**Conclusions:**

This study offers a helpful starting point in identifying opportunities to address a critical service delivery gap in India. Given the existing burden on the public health and health systems, scale-up will require innovative, resource-appropriate solutions. The findings also point to the need to center equity in the design and scale-up of tobacco cessation supports so that marginalized and underserved groups will have equitable access to these critical services.

**Supplementary Information:**

The online version contains supplementary material available at 10.1186/s12889-022-14538-2.

## Background

India is home to about 12% of the world's tobacco users, with tobacco use and exposure linked to about 1.35 million deaths each year [1, 2]. Popular smokeless tobacco products include khaini (a tobacco/lime blend), pan (bits of lime, areca nut, and spices in a betel leaf), gutka/pan masala (a mixture of crushed lime and areca nut), and mishri (a tobacco product used as dentifrice). Smoked tobacco is often consumed in the form of bidis (a traditional form of cigarettes), cigarettes, and cigars [1, 3]. Men, rural residents, people with no formal education, daily wage laborers, and people experiencing material deprivation are more likely to use smoked or smokeless tobacco products and suffer disproportionately higher morbidity and mortality related to tobacco use [4]. It is critical to find scalable solutions to address tobacco use among users of low socioeconomic status, as they typically experience significant barriers to care, often leading to catastrophic expenditures and unresolvable debt [5, 6]. The Government of India has enacted several tobacco control policies to restrict tobacco advertising, sales, and use while also increasing health promotion and cessation service offerings through tobacco cessation centers [7–9]. These efforts, along with others, have reduced tobacco use rates from 35% of adults in 2009-2010 to 29% in 2016-2017. However, this still leaves 267 million current tobacco users who could benefit from services [10, 11]. As a complement to policy and systems change, cessation services are a critical component of the national tobacco control program. National data from India in 2017 suggest that about half of tobacco users are interested in quitting. Among those who had made quit attempts, almost 70% did so independently. Among those who visited a healthcare provider in the previous 12 months, about one-half of smokers and one-third of smokeless tobacco users reported receiving advice to quit from a healthcare provider [11]. The insufficient delivery of cessation supports is the focus of our attention in this study.

In India, there are critical challenges to delivering tobacco control interventions that are standard in high-income countries. The programs usually rely on credentialed staff and pharmacotherapy, which is currently unaffordable for most people in India and may or may not be effective with smokeless tobacco users [9, 12, 13]. For these reasons, it is worth exploring other less intensive solutions, such as brief advice interventions. These interventions involve screening patients for tobacco use, advising them to quit, and referring them to cessation resources [14]. Brief advice interventions can typically be administered in 30 seconds to 2 minutes and are effective for tobacco control [15, 16]. Brief advice given by health care providers increases quit attempts and is associated with increased use of counseling and other cessation supports [16, 17]. A recent review of tobacco cessation interventions in low- and middle-income countries (LMICs) found that brief advice interventions were more effective than standard education at promoting cessation [18]. Indeed, adding deeper motivational counseling and pharmacotherapy can increase the impact on individuals. Still, given resource constraints in the Indian health sector, it is imperative to find low-cost, scalable solutions. Simple forms of these brief advice programs can result in 1-3% increased cessation rates over no advice, which could translate to 2.7 – 8.0 million fewer tobacco users in India [11, 19, 20].

While brief advice interventions hold promise, they require further examination due to four disconnects. First, most evidence on brief advice interventions comes from high-income, Western countries with dramatically different health systems and resource availability [21, 22]. Second, most of the evidence on brief advice relates to smoked tobacco. However, in India, the prevalence of smokeless tobacco use is almost twice that of smoked tobacco (22% vs. 11% of the overall population) [3]. A systematic review of smokeless tobacco cessation interventions suggests that behavioral interventions can be effective for smokeless tobacco users [21]. Third, we must consider the potential for brief advice interventions within the Indian healthcare system. In India, the private sector provides the bulk of care, accounting for 78% of health expenditures, and patients commonly pay for services out-of-pocket (71% of total health spending). As a result, low-income households experience significant barriers to care and are often at risk for catastrophic expenditures and unresolvable debt [5, 6]. Finally, we must center equity in considerations of offering brief advice services. Healthcare services in India are patterned by rural vs. urban residence, given that 59% of health workers and 80% of specialists are based in urban areas, yet only 28% of the population lives in urban areas [23]. Additionally, healthcare access in India is also patterned by sociodemographics, with lower access among women, groups of lower socioeconomic status, and members of scheduled castes and tribes [5].

As part of a broader exploration of the potential for brief advice to offer scalable tobacco cessation in India, we conducted the present study to examine the relationships between demographic and socioeconomic characteristics and being asked about tobacco use and/or advised to quit. While we cannot assess the duration of interactions for being asked and/or advised in these data, they point to the general rate of light-touch tobacco control engagement that may be amenable to intervention. Our two core questions were: 1) among the general population, what percentage of tobacco users are asked about tobacco use and offered cessation advice by a healthcare provider? and 2) are marginalized and underserved groups (as indicated by wealth, rural vs. urban residence, caste, and education levels) less likely to be asked by a healthcare provider about tobacco use and advised to quit?

## Methods

### Background on GATS-2 Data

Data for this study come from India's 2016-2017 Global Adult Tobacco Survey – 2^nd^ round (GATS-2). Although researchers have analyzed GATS-2 to understand the link between demographic and socioeconomic factors and tobacco use, quit attempts, and willingness to quit [4, 24–26], we are unaware of a study examining the link between these factors and the extent to which individuals are asked or advised about tobacco use. GATS is a standardized, nationally representative survey used in many nations to measure tobacco use and control among adults. In India, the first round of GATS was conducted in 2009-2010, and the second (referred to as GATS-2) in 2016-2017 [27]. GATS-2 surveyed non-institutionalized individuals aged 15 and older in all states and territories of India. It measured factors such as individual background characteristics, tobacco use, cessation attempts, being asked about tobacco and being advised to quit (referred to as receipt of “ask and advise” services), media exposure related to tobacco products, the economics of tobacco use, exposure to secondhand smoke, and tobacco-related knowledge, attitudes, and perceptions [11].

Participation was voluntary, and the initiative used a three-stage sampling design for urban areas and a two-stage sampling design for rural areas. The survey is divided into a household questionnaire and an individual questionnaire. The head of the household (or another adult member if the head was not present) completed the household questionnaire, and then a member of the household aged 15 and older was randomly selected to complete the individual questionnaire. A sample of 84,047 households was selected for the survey, and of those, 77,170 households (92%) and 74,037 individuals participated [27]. This analysis focuses on the data from individuals.

### Measures

The 74,037 individuals who completed the questionnaires were asked about their past and current use of tobacco products. In the questionnaire, smoked tobacco included *bidis, cigarettes, cigars, cheroots, rolled cigarettes, tobacco rolled in maize leaf and newspaper, hukkah, pipes, chillum, chutta*—not including electronic cigarettes or smokeless tobacco. For smokeless tobacco (which is sniffed through the nose, held in the mouth, or chewed), the survey specified *tobacco leaf, betel quid with tobacco, sada/surti, khaini or tobacco lime mixture, gutkha, paan masala with zarda, mawa, gul, gudaku, mishri.* Current use of tobacco products was assessed based on the questions, "Do you currently smoke tobacco on a daily basis, less than daily, or not at all?" and "Do you currently use smokeless tobacco on a daily basis, less than daily, or not at all?" Those who responded that they use smoked tobacco or smokeless tobacco on a daily basis were included in this analysis. A separate set of questions asked respondents if they had been seen by a healthcare provider in the last year. Those with a visit in the last year received the question "Were you asked if you smoke or use smokeless tobacco?" If yes, respondents were asked if had been advised to quit smoking tobacco or stop using smokeless tobacco by their health care provider.

We calculated the prevalence of being asked about tobacco use and advised to quit at a national level for daily users of smoked or smokeless tobacco. We used demographic and socioeconomic factors as the predictor variables to assess their associations with provider interactions. In our analyses, we included the following demographic variables: residence status (urban or rural); age group (15-24, 25-44, 45-64, or 65+); gender (male or female); education (no formal schooling; less than primary school completed; primary school completed; secondary school completed; higher secondary; college or above); caste (scheduled caste, scheduled tribe, other backward caste, or others). For readers unfamiliar with the Indian caste system, access to education, healthcare, and other opportunities are patterned by these social identities. Scheduled castes and tribes are individuals from marginalized, typically lower socioeconomic status (SES) groups, who typically have poorer access to healthcare services than their counterparts. Individuals in the "other backward caste" group typically have higher SES than scheduled castes and scheduled tribes, with the "others" group being a heterogeneous group that includes, but is not limited to, the highest SES castes [28]. In addition, we followed a previously used approach of applying principal component analysis to derive a wealth index based on various household possessions (electricity, flush toilet, cell phone, television, car, washing machine, computer/laptop, internet connection, air conditioner, and electric fan) and categorized participants into quintiles (poorest, poor, middle, wealthier, or wealthiest) [29].

### Data analysis

We conducted descriptive analyses to assess the weighted prevalence by demographic and socioeconomic factors and usage patterns of tobacco products separately by smoked tobacco or smokeless tobacco and dual use. After stratifying by tobacco type among daily tobacco users (*N*=12,721 daily users of smokeless tobacco; *N*=7,647 daily users of smoked tobacco), we used Chi-square tests to assess the bivariate associations between demographic predictors and participants' interactions with their healthcare providers in the past 12 months concerning being asked and advised about tobacco use. This analysis was restricted to daily users of smoked or smokeless tobacco, the predominant group of current tobacco users in India and therefore, the priority targets for brief advice and other tobacco cessation supports [11]. The other survey response option for current users was "less than daily," which is too broad of a group to be confident they warrant intervention. We note that dual users of smoked and smokeless tobacco were not excluded from the analysis. They will still present as daily users to healthcare providers and were too small of a group to support separate analyses.

We further examined the correlations among the predictor variables (e.g., Spearman correlations among ordinal predictors) to ascertain potential collinearity among predictors (Supplemental Table 1). Finally we conducted multivariate logistic regression to assess the association of demographic and socioeconomic conditions with participants' receipt of “ask and advise services.” For our analyses, we used the survey weight that accounted for the design base weight and response rate differences for households and individuals in the GATS-2 survey. We performed all statistical analyses using software SAS version 9.4 with a p-value of less than 0.05 for statistical significance. This study was conducted by university- and practice-based researchers. The academics have rich expertise with tobacco control, public health in India, health equity, and health communication. The practice-based researchers have rich expertise conducting tobacco control interventions for low-resource settings in Maharashtra and several other Indian states. Both university- and practice-based researchers have co-authored this piece.

## Results

The nationally weighted data for the GATS-2 survey describe a predominantly rural sample (about 65%) that skews towards the middle age bracket of ages 25 to 44 (Table 1). Almost two-thirds of the sample (64%) had a primary school education or no formal schooling. About a quarter of the sample used tobacco daily, with the bulk of the daily users consuming smokeless tobacco. Rates of being asked and advised to quit among daily tobacco users were 22% and 19%, respectively.

**Table 1:** Demographic characteristics of nationally representative sample of Indian population ages 15 and older regarding tobacco use, from Global Adult Tobacco Survey (GATS-2) (2016-2017) *n*=74,037

Variable	Characteristics	National Weighted Prevalence (%)
Residence status	Urban	34.49
	Rural	65.51
Age (years)	15-24	26.81
	25-44	41.31
	45-64	23.46
	65+	8.41
Gender	Male	51.10
	Female	48.90
Wealth in quintiles^a^	Poorest	24.16
	Poor	17.89
	Middle	23.76
	Wealthier	19.28
	Wealthiest	14.92
Education^b^	No formal schooling, less than primary school completed	35.66
	Primary school completed	28.18
	Secondary school completed	14.08
	Higher secondary	11.16
	College or above	10.92
Caste	Scheduled Caste	19.09
	Scheduled Tribe	8.87
	Other Backward Caste	45.27
	None of the above	26.77
Current use of smoked tobacco	Daily	8.59
	Less than daily	2.09
	Not at all	89.33
Current use of smokeless tobacco	Daily	18.24
	Less than daily	3.14
	Not at all	78.62
Current use of smoked tobacco AND smokeless tobacco	Daily	1.91
	Less than daily	1.54
	Not at all	96.56
Current use of smoked tobacco OR smokeless tobacco	Daily	24.92
	Less than daily	3.69
	Not at all	71.39
Quit attempt made in the past 12 months, current smoked tobacco users^c^	Yes	36.36
	No	63.64
Quit attempt made in the past 12 months, current smokeless tobacco users^d^	Yes	32.01
	No	67.99
Asked about tobacco use by a healthcare provider in the past 12 months, current tobacco users^e^	Yes	21.53
	No	78.47
Advised to quit tobacco by a healthcare provider in the past 12 months, among current tobacco users^e^	Yes	18.68
	No	81.32
Asked about tobacco use by a healthcare provider in the past 12 months, among current smoked tobacco users^f^	Yes	27.13
	No	72.87
Advised to quit tobacco by a healthcare provider in the past 12 months, among current smoked tobacco users^f^	Yes	24.29
	No	75.70
Asked about tobacco use by a healthcare provider in the past 12 months, among current smokeless tobacco users^g^	Yes	17.73
	No	82.26
Advised to quit tobacco by a healthcare provider in the past 12 months, among current smokeless tobacco users^g^	Yes	15.02
	No	84.98

^**a**^*N*=73,299

^**b**^*N*=73,978

^**c**^*N*=9,490

^**d**^*N*=15,225

^**e**^*N*=21,857

^**f**^*N*=9,499

^**g**^*N*=15,235

We then turned to the relationships between sociodemographic factors and being asked or advised about tobacco use. We analyzed data from the 12,721 daily users of smoked or smokeless tobacco. As highlighted in Table 2, we found significantly lower rates of being asked about tobacco for people who were younger and had lower wealth. We also found significant differences by caste, with markedly lower rates of being asked among members of scheduled tribes than other groups. Again, scheduled tribes and scheduled castes are marginalized and underserved in India. Statistically significant differences were not observed based on education level, gender, or residence. These patterns held for rates of being advised to quit tobacco, though members of "other backward castes" also had low reports of being advised to quit tobacco by a healthcare provider.

**Table 2:** Chi-square tests for associations of demographic factors with being asked and advised about tobacco by a healthcare provider among daily users of smokeless tobacco, national weighted data (*n*=12,721)

	Asked about tobacco use by a healthcare provider in the past 12 months		Advised to quit tobacco by a healthcare provider in the past 12 months	
Socio demographic factors	Yes (%)	Chi-square test (*p*-value)	Yes (%)	Chi-square test (*p*-value)
**Residence**		2.15 (0.1424)		1.83 (0.1761)
Urban	19.35		16.34	
Rural	17.17		14.47	
**Age (years)**		18.41 (0.0004)		17.80 (0.0005)
15-24	13.27		10.74	
25-44	16.39		13.98	
45-64	20.70		17.80	
65+	19.04		14.98	
**Gender**		0.05 (0.8202)		0.04 (0.8378)
Female	17.88		15.07	
Male	17.62		14.85	
**Wealth in quintiles**^**a**^		13.79 (0.0080)		11.64 (0.0202)
Poorest	15.26		12.96	
Poor	18.07		15.19	
Middle	19.16		16.35	
Wealthier	19.09		15.54	
Wealthiest	22.94		19.61	
**Education**^**b**^		1.97 (0.7415)		0.96 (0.9155)
No formal schooling, less than primary school completed	17.37		14.60	
Primary school completed	18.35		15.59	
Secondary school completed	18.01		14.78	
Higher secondary	15.25		13.87	
College or above	19.86		15.70	
**Caste**		21.92 (<0.0001)		16.74 (0.0008)
Scheduled Caste	17.49		14.37	
Scheduled Tribe	12.12		15.07	
Other Backward Caste	19.86		10.44	
None of the above	17.27		16.59	

^**a**^*N*=12,594

^**b**^*N*=12,714

As summarized in Table 3, we repeated the examination of the association between demographic, place, and socioeconomic factors and being asked or advised about tobacco use among daily users of smoked tobacco. Once more, we found significantly lower rates of being asked about tobacco for people who were younger and had lower wealth. We also found significant differences by caste, with lower rates of being asked among members of scheduled tribes than other groups. No statistically significant differences were observed based on rural vs. urban residence, gender, or education level. The same pattern held for receipt of advice to quit tobacco use. In comparing Tables 3 and 4, we see a consistent pattern in which smoked tobacco users reported higher rates of being asked and advised about tobacco use than smokeless tobacco users.

**Table 3:** Chi-square tests for associations of demographic factors with being asked and advised about tobacco by a healthcare provider among daily users of smoked tobacco, national weighted data (*n*=7,647)

	Asked about tobacco use by a healthcare provider in the past 12 months		Advised to quit tobacco by a healthcare provider in the past 12 months	
Socio demographic factors	Yes (%)	Chi-square test (*p*-value)	Yes (%)	Chi-square test (*p*-value)
**Residence**		0.16 (0.6859)		0.003 (0.9574)
Urban	29.73		25.83	
Rural	28.85		25.94	
**Age (years)**		32.48 (<0.0001)		40.70 (<0.0001)
15-24	19.24		12.99	
25-44	24.42		21.71	
45-64	33.40		30.07	
65+	34.53		31.78	
**Gender**		1.33 (0.2481)		2.14 (0.1436)
Female	25.88		22.08	
Male	29.41		26.31	
**Wealth in quintiles**^**a**^		12.96 (0.0115)		9.12 (0.0583)
Poorest	26.10		23.16	
Poor	27.64		25.85	
Middle	30.82		27.41	
Wealthier	31.96		27.13	
Wealthiest	36.58		32.21	
**Education**^**b**^		2.67 (0.6143)		4.55 (0.3372)
No formal schooling, less than primary school completed	30.02		27.44	
Primary school completed	27.71		23.67	
Secondary school completed	29.19		24.95	
Higher secondary	25.14		23.49	
College or above	31.04		26.87	
**Caste**		8.66 (0.0342)		4.98 (0.1735)
Scheduled Caste	28.40		25.81	
Scheduled Tribe	22.27		20.80	
Other Backward Caste	29.47		26.02	
None of the above	31.86		27.95	

^**a**^*N*=7,584

^**b**^*N*=7,645

**Table 4:** Associations of being asked about and advised to quit tobacco use by a healthcare provider in the past 12 months with individual's sociodemographic factors among daily users of smoked or smokeless tobacco, multivariate logistic regression (*n*=18,744)

	Asked about tobacco use by a healthcare provider in the past 12 months (Multivariate)		Advised to quit tobacco by a healthcare provider in the past 12 months (Multivariate)	
Socio demographic factors	AOR^a^ (95% CI±)	*p*-value (values ≤ 0.05 are bolded)	AOR (95% CI)	*p*-value (values ≤ 0.05 are bolded)
**Residence**				
Urban	1.00 (0.84, 1.19)	0.9973	0.98 (0.82, 1.18)	0.8564
Rural	Ref.		Ref.	
**Age**				
15-24	Ref.		Ref.	
25-44	1.35 (1.00, 1.82)	**0.0488**	1.55 (1.12, 2.13)	**0.0081**
45-64	1.94 (1.44, 2.61)	**<0.0001**	2.25 (1.63, 3.10)	**<0.0001**
65+	1.92 (1.41, 2.63)	**<0.0001**	2.09 (1.49, 2.94)	**<0.0001**
**Gender**				
Female	Ref.		Ref.	
Male	1.39 (1.20, 1.60)	**<0.0001**	1.44 (1.24, 1.68)	**<0.0001**
**Wealth in quintiles**				
Poorest	Ref.		Ref.	
Poor	1.20 (1.03, 1.41)	**0.0224**	1.25 (1.05, 1.48)	**0.0100**
Middle	1.27 (1.07, 1.50)	**0.0060**	1.31 (1.10, 1.57)	**0.0030**
Wealthier	1.37 (1.10, 1.70)	**0.0051**	1.32 (1.06, 1.65)	**0.0139**
Wealthiest	1.73 (1.34, 2.24)	**<0.0001**	1.78 (1.36, 2.33)	**<0.0001**
**Education**				
No formal schooling, less than primary school completed	Ref.		Ref.	
Primary school completed	0.99 (0.86, 1.14)	0.9037	0.96 (0.83, 1.11)	0.5682
Secondary school completed	0.86 (0.70, 1.07)	0.1820	0.81 (0.64, 1.01)	0.0650
Higher secondary	0.70 (0.51, 0.96)	**0.0282**	0.75 (0.54, 1.05)	0.0895
College or above	0.91 (0.64, 1.29)	0.5834	0.86 (0.60, 1.23)	0.4101
**Caste**				
Scheduled Caste	1.03 (0.86, 1.24)	0.7387	1.07 (0.88, 1.29)	0.5104
Scheduled Tribe	0.71 (0.58, 0.88)	**0.0017**	0.76 (0.61, 0.96)	**0.0186**
Other Backward Caste	1.07 (0.92, 1.26)	0.3817	1.08 (0.91, 1.28)	0.3802
None of the above	Ref.		Ref.	

^a^*AOR* Adjusted odds ratio; ± *CI* Confidence interval

Our final analyses used a regression model to examine the associations of demographic, rural vs. urban residence, and socioeconomic characteristics with 1) being asked about tobacco and 2) being advised to quit. We first examined correlations among education, wealth, caste, rural vs. urban residence, age, and gender. The correlations of considerable magnitude included: education-wealth (0.49), and rural-wealth (-0.45). However, given all of the Spearman correlations were below 0.6, we retained all of the variables in the adjusted analysis. As presented in Table 4, we found that the odds of being asked about tobacco use by a healthcare provider was lower for younger, female, and less wealthy individuals. Those with higher secondary education and members of scheduled tribes also had lower odds of being asked about tobacco use. For receipt of advice, we found lower odds among younger, female, and less wealthy individuals. Members of scheduled tribes also reported lower odds of being advised to quit tobacco use.

## Discussion

This analysis sought to characterize the rate and social patterning of tobacco users in India being asked about tobacco use and advised to quit. Broadly, the results highlight low rates of "ask and advise" services and suggest tremendous room for intervention. There is a clear need to address existing inequities based on age, wealth, and caste in attempts to implement and scale-up tobacco control offerings. Nationally, among daily tobacco users, we found low rates of individuals reporting being asked about tobacco use or advised to quit by a healthcare provider. The opportunity to address this service gap is emphasized by World Health Organization guidance, which notes that routinization of "ask and advise" services by primary care providers could reach over 80% of all tobacco users every year and support 40% of tobacco users to make a quit attempt and 2-3% to be successful [30]. Given the large numbers of tobacco users in India, this could translate to 5.3-8.0 million tobacco users in India successfully quitting per year. As one of many strategies in a comprehensive tobacco control portfolio, such an addition can be valuable.

These findings also add to existing GATS-2 analyses, which have focused exclusively on tobacco users who have visited a healthcare provider. Among those who visited a healthcare provider in the previous 12 months, about 49% of smokers and 32% of smokeless tobacco users were advised to quit [11]. The lower rates of being asked and advised we found among all users are consistent with a recent report from the Indian National Institute of Mental Health and Neuro Sciences estimates the treatment gap for tobacco, or the proportion of users who are not receiving cessation services, at 92% [31]. This is also consistent with broader data from LMICs. In 2015, a survey of signatories to the World Health Organization Framework Convention on Tobacco Control found that fewer than half of the countries had integrated brief cessation advice into existing services (44%) with an even lower rate of 31% among LMICs [32]. Interestingly, smoked tobacco users reported higher rates of ”ask and advise” services than users of smokeless tobacco. This may be a function of local and regional norms in which smokeless tobacco is more socially acceptable and is often perceived to be less harmful than smoked tobacco [33, 34].

In addition to low overall rates of being asked about tobacco use or advised to quit, we found that rates of service receipt were patterned by age, gender, wealth, education, and caste in our final regression model. This reflects broader trends in healthcare access in India, with limited access among rural residents, women, groups of lower socioeconomic status, and members of scheduled castes and tribes [5, 23]. Specific to tobacco, these findings echo an analysis of the first round of GATS data from India (2009-2010), which found that older and male tobacco users who visited a healthcare provider were more likely to be screened for tobacco use than their counterparts [35]. The age-related gap is critical, given that about 12% of young people aged 15-24 in India were using tobacco and the average age of initiation was 19. Tobacco habits are typically established in adolescence and have long-term impacts on cardiovascular disease, cancer, and other tobacco-related diseases [36]. Our findings are also consistent with the existing literature from India and other LMICs. An analysis of GATS data from 17 countries, including India, found that older smoked tobacco users were more likely to be asked and advised than younger users in 14 out of the 17 countries [37]. This may be attributed in part to the fact that older people visit healthcare providers and generally use healthcare services more frequently than those in the younger age groups [38]. The patterning of women reporting less "ask and advise" services than men is consistent with the overall lower utilization of healthcare services by women in India [39]. Providers may also be less likely to ask and advise women about tobacco, given that they tend to have lower tobacco usage rates in India [4]. Lower reports of services among individuals with less wealth are consistent with a broader pattern in India. Part of the challenge with the routinization of this type of preventive service is the reliance on the private sector (78% of health expenditures) and a system in which patients typically pay out-of-pocket (71% of health spending) [5]. Similar patterns have been found with other preventive services, such as screening for breast and cervical cancer [40, 41]. The association between caste and receipt of “ask and advise” services is consistent with broader patterns of scheduled castes and scheduled tribes having lower access to healthcare services (including preventive services) and bearing a disproportionate burden of morbidity and mortality [42]. The social exclusion and discrimination based on caste pose important challenges to future efforts to scale brief advice interventions equitably [28]. Interestingly, our results did not demonstrate an extensive education-based differential in receipt of services. This is a contrast to other work demonstrating the impact of education (independent of wealth) on patterning of health inequities in India [43].

Taken together, these findings highlight both the need to improve overall "ask and advise" services in India, and also emphasize the importance of an equity-promoting perspective when it comes to allocation of services. This must take place in the context of overall distribution of disease burden, e.g., rural men being the subpopulation most likely to suffer from tobacco-related cancers [44]. Grounding efforts in critical public health theoretical approaches [45] or equity-focused frameworks and processes [46, 47] will support the advancement of health equity goals in low-resource settings.

We place our results in the context of a set of limitations and strengths. First, the data collected in GATS-2 were based on individuals' self-report, and GATS-2 data do not contain information about local or regional norms, intervention programs, and tobacco use and treatment policies that may influence use and receipt of “ask and advise” services. Second, we analyzed reports from daily tobacco users, excluding individuals who report using tobacco less often than daily. A portion of this subgroup may benefit greatly from being asked and advised about tobacco use, but given the potential variation in the group, we excluded them from this analysis. Additionally, the report of advice from a healthcare practitioner could mean a wide range of things, some evidence-based (e.g., the 5A's [30]) and some that could cause no impact or even harm. Moreover, since this study is assessing the gap with provider-administered services and support for tobacco cessation, those who did not visit a healthcare provider are not included in the analyses. Future investigation is warranted to better understand how to address those who use tobacco but do not have access to healthcare providers. Related to our interest in brief interventions as a potential solution, these data do not contain any details regarding the length of the interaction. Finally, we assessed data at the national level, while acknowledging that there is tremendous variation between regions of the country, as well as between high- and low-development areas [48]. At the same time, a series of important strengths outweigh these limitations. First, these data come from a recent, nationally representative sample and focus on the overall population of daily tobacco users (versus only those who have visited a healthcare provider in the past year). Second, the data examine a wide range of potential sociodemographic determinants that could impact the receipt of preventive services. Finally, this study builds the literature related to brief advice among LMICs and users of smokeless tobacco, a gap that has previously limited the broader application of these interventions in India and in other countries with large populations who use smokeless tobacco.

## Conclusions

Our study highlights the need to address the current service gap related to “ask and advise” services offered by healthcare provider. We recognize the limited human resources for health in India and the projection that the country's cancer burden will increase from 1 to 1.75 million new cases per year between 2012 and 2035 [48]. Thus, future work must examine ways to deliver “ask and advise” services without further taxing an already overburdened healthcare system. The wide range of national health programs in India offers a diverse set of targets for integrating brief advice services. An important advantage is that these services do not require specialized equipment or facilities and can be delivered by providers who do not have formal medical credentials but are more readily available than highly-credentialed providers, utilizing a task-shifting approach. With this work, capacity-building and resources for a wide range of service delivery professionals are the core required supports. Policies to increase access with an explicit equity-promoting focus will be required to address the social patterning of access to “ask and advise” services. For example, direction of resources and supports for rural providers and those working with scheduled tribes will likely be needed to advance equity goals. In these ways, brief interventions for tobacco use can complement policy and other strategies to reduce tobacco use in India and address a major driver of disease burden.

## Supplementary Information

**Additional file 1.**

## Data Availability

The datasets analyzed during the current study are available in the CDC repository, https://nccd.cdc.gov/GTSSDataSurveyResources/Ancillary/DataReports.aspx?Country=180&CAID=2&Survey=4&WHORegion=2&Site=3840002016

## Abbreviations

GATS-2: Global Adult Tobacco Survey
LMIC: low- and middle-income country
SES: Socioeconomic Status
AOR: Adjusted odds ratio
CI: Confidence interval
