# Supplementary material for: Associations between sociodemographic factors and receiving "ask and advise" services from healthcare providers in India: analysis of the national GATS-2 dataset
Source: BMC Public Health. 2022 Nov 18;22:2115. doi: 10.1186/s12889-022-14538-2 (PMC9673333; doi:10.1186/s12889-022-14538-2)
Supplement: Supplementary file 1 — Additional file 1. [file 12889_2022_14538_MOESM1_ESM.docx]

|  | **Urban** | **Age 25-44** | **Age 45-64** | **Age 65+** | **Male** | **Wealth 2^nd^** | **Wealth 3^rd^** | **Wealth 4^th^** | **Wealth 5^th^** | **Edu, primary** | **Edu, secondary** | **Edu, higher secondary** | **Edu, college or above** | **Scheduled caste** | **Scheduled tribes** | **Other backward class** |
| --- | --- | --- | --- | --- | --- | --- | --- | --- | --- | --- | --- | --- | --- | --- | --- | --- |
| **Urban** | - | 0.017* | 0.008* | -0.0225* | -0.0153* | -0.1554* | -0.0278* | 0.1331* | 0.3548* | -0.0355* | 0.0508* | 0.0841* | 0.2253* | -0.062* | -0.134 | 0.0401* |
| **Age 25-44** |  | - | -0.568* | -0.286* | -0.010* | 0.0154* | 0.0119* | 0.002 | -0.017 | 0.0445* | -0.001 | -0.007* | 0.074* | 0.016* | 0.020* | -0.013* |
| **Age 45-64** |  |  | - | -0.175* | 0.007 | -0.017* | -0.008* | -0.0004 | 0.0325* | -0.042* | -0.055* | -0.090* | -0.053* | -0.021* | -0.023* | 0.018* |
| **Age 65+** |  |  |  | - | 0.016* | -0.013* | -0.019* | -0.011* | 0.002 | -0.066* | -0.060* | -0.0703* | -0.0543* | -0.011* | -0.028 | 0.009* |
| **Male** |  |  |  |  | - | 0.011* | 0.031* | -0.029* | -0.031* | 0.049* | 0.060* | 0.054* | 0.053* | -0.013* | 0.0228* | 0.0105* |
| **Wealth 2^nd^** |  |  |  |  |  | - | -0.237* | -0.232* | -0.236* | 0.071* | -0.034* | -0.062* | -0.127* | 0.043* | 0.073* | -0.022* |
| **Wealth 3^rd^** |  |  |  |  |  |  | - | -0.250* | -0.254* | 0.059* | 0.021* | -0.011* | -0.086* | 0.020* | -0.046* | 0.071* |
| **Wealth 4^th^** |  |  |  |  |  |  |  | - | -0.249* | 0.017* | 0.079* | 0.068* | 0.0203* | -0.013* | -0.081* | 0.035* |
| **Wealth 5^th^** |  |  |  |  |  |  |  |  | - | -0.123* | 0.053* | 0.137* | 0.364* | -0.093* | -0.097* | -0.043* |
| **Edu. primary** |  |  |  |  |  |  |  |  |  | - | -0.253* | -0.218* | -0.2302* | 0.006* | 0.037* | -0.005* |
| **Edu, secondary** |  |  |  |  |  |  |  |  |  |  | - | -0.134* | -0.147* | -0.027* | -0.023* | 0.006* |
| **Edu, higher secondary** |  |  |  |  |  |  |  |  |  |  |  | - | -0.1271* | -0.032* | -0.026* | -0.012* |
| **Edu, college or above** |  |  |  |  |  |  |  |  |  |  |  |  | - | -0.072* | -0.055* | -0.017* |
| **Scheduled caste** |  |  |  |  |  |  |  |  |  |  |  |  |  | - | -0.203* | -0.351* |
| **Scheduled tribes** |  |  |  |  |  |  |  |  |  |  |  |  |  |  | - | -0.338* |
| **Other backward class** |  |  |  |  |  |  |  |  |  |  |  |  |  |  |  | - |

Supplemental Table 1. Correlations (Spearman) between regression predictor variables

* Significant correlations between variables at the p < 0.05 level.
